# Genetic diversity and conservation of Siberian apricot (*Prunus sibirica* L.) based on microsatellite markers

**DOI:** 10.1038/s41598-023-37993-2

**Published:** 2023-07-11

**Authors:** Xinxin Wang, Li Wang, Yongqiang Sun, Jianhua Chen, Quangang Liu, Shengjun Dong

**Affiliations:** 1 College of Forestry, Shenyang Agricultural University, Shenyang, 110866 Liaoning China; 2 Key Laboratory for Silviculture of Liaoning Province, Shenyang, 110866 Liaoning China

**Keywords:** Forestry, Genetics, Plant genetics, Population genetics, Plant sciences, Natural variation in plants, Plant genetics, Biodiversity, Conservation biology, Forestry

## Abstract

Siberian apricot (*Prunus sibirica* L.) is a woody tree species of ecological, economic, and social importance. To evaluate the genetic diversity, differentiation, and structure of *P. sibirica*, we analyzed 176 individuals from 10 natural populations using 14 microsatellite markers. These markers generated 194 alleles in total. The mean number of alleles (13.8571) was higher than the mean number of effective alleles (6.4822). The average expected heterozygosity (0.8292) was higher than the average observed heterozygosity (0.3178). Shannon information index and polymorphism information content were separately 2.0610 and 0.8093, demonstrating the rich genetic diversity of *P. sibirica*. Analysis of molecular variance revealed that 85% of the genetic variation occurred within populations, with only 15% among them. The genetic differentiation coefficient and gene flow were separately 0.151 and 1.401, indicating a high degree of genetic differentiation. Clustering results showed that a genetic distance coefficient of 0.6 divided the 10 natural populations into two subgroups (subgroups A and B). STRUCTURE and principal coordinate analysis divided the 176 individuals into two subgroups (clusters 1 and 2). Mantel tests revealed that genetic distance was correlated with geographical distance and elevation differences. These findings can contribute to the effective conservation and management of *P. sibirica* resources.

## Introduction

Siberian apricot (*Prunus sibirica* L.) is a deciduous perennial woody tree species of the genus *Prunus* in the Rosaceae subfamily Prunoideae^1^. Given its white or pinkish flowers in spring, ripe yellow fruit in summer, and highly diverse leaf colors in autumn, the tree is considered to have particular ornamental value^2,3^. As a pioneer tree species in the restoration of vegetation, *P. sibirica* can tolerate cold, drought, and barren land and, thus, has high ecological value^1,4^. In addition, the seeds of this species contain amygdalin and high-quality oils, which have significant economic value^5,6^. Consequently, the government in north China promotes this tree as an economic forest species in semi-arid and arid areas, and breeding improved varieties with excellent characteristics is desirable.

As a source of genetic variation and potentially valuable agronomic traits, wild genetic resources can make a contribution to crop improvement^7^. *P. sibirica* is native to northern and northeastern China and has distribution in its neighboring countries of Russia and Mongolia^8,9^. Increased study and introduction of these wild germplasm resources will facilitate breed new varieties with improved characteristics. Such improvement is based on the germplasm collection, assessment, and conservation of genetic resources^10^. To date, reports on *P. sibirica* germplasm resources have been limited to parts of northern and northeastern China^11–16^. However, those populations located in less favorable and more peripheral habitats are also significant with unique adaptive genetic diversity^17^. Our incomplete understanding of these populations could limit the efficient utilization of *P. sibirica* genetic resources^10,18^. Furthermore, human interference is increasingly threatening the survival of these natural populations^13,19^, and the resulting habitat fragmentation will reduce the occurrence of genetic variation and the evolutionary potential of *P. sibirica*^20^. Given this scenario, it is urgent to devise and implement protection strategies designed for the sustainable protection of the wild resources of *P. sibirica*.

Assessing the genetic diversity of *P. sibirica* genetic resources is particularly important for breeding and conservation programs^21^. In the development of superior varieties, genetic diversity can reflect the improvement potential of species in the breeding process^22^. Moreover, genetic diversity is fundamental to sustaining the adaptive capacities of species, the loss of which can potentially compromise the survival potential of affected populations or species^23^. The distribution pattern of genetic diversity within a species is an essential reference for designing conservation measures^24^. In this respect, genetic markers can reflect differences in DNA sequences and are commonly applied to study the amount and distribution of genetic variation^25^. Compared with morphological and biochemical markers, molecular markers can identify variation at specific loci and assist in quantifying genetic diversity levels^22^. Among the various kinds of molecular markers, microsatellite markers have the favorable characteristics of co-dominant inheritance, high polymorphism, good repeatability, and regular distribution^26,27^. Consequently, microsatellite markers are widely applied to detect the genetic diversity of plants^28–31^, particularly in genus *Prunus*^21,32–34^.

In view of these considerations, we obtained genetic materials from 176 samples in 10 natural *P. sibirica* populations and applied 14 pairs of microsatellite markers to assess the level of genetic diversity. The nuclear of our research were to (1) assess the genetic diversity, differentiation, and structure of *P. sibirica* and (2) develop strategies to protect *P. sibirica* germplasm resources. The results of our research will contribute to the effective conservation and management of the wild *P. sibirica* resources.

## Materials and methods

### Plant materials

A total of 176 samples collected from 10 natural populations of *P. sibirica* were used in this study. Location information for 176 samples (Table S1) was obtained using GPS. With the exception of population R, distributed in the Zabaykalsky Krai region of Russia, all other populations are distributed in northeast China. The sampled populations cover a broad geographical span and ecological spectrum, with longitudes ranging from N 40°01′ to 53°05′, latitudes from 115°23′ to 123°01′, and elevations from 222 to 1292 m. We collected the fresh young leaves of trees in the field, froze them in liquid nitrogen, and stored them at − 80 °C until use.

### DNA extraction and amplification

We extracted the total genomic DNA from 176 samples individually using the Plant Genome DNA Extraction Kit (TianGen, Beijing, China) and purified them by 1% agarose gel electrophoresis. NanoDrop2000 spectrophotometry (Thermo Fisher Scientific, Waltham, MA, USA) was used to test the concentration and purity of the extracted DNA, and the high quality DNA was stored at − 20 °C until use.

In our previous studies, we developed microsatellite markers for *P. sibirica*^12,35^. We used 14 pairs of microsatellite markers (Table 1) to amplify the extracted genomic DNA. PCR amplifications were performed in 20-µL mixtures comprising 20 ng template DNA, 0.25 mmol/L dNTPs, 2 mmol/L Mg^2+^, 1 U Taq polymerase, 10 × PCR buffer, and 0.15 µmol/L primers. Amplification was performed by the following parameters: (1) initiated with a 105 °C hot lid, (2) initially denatured at 94 °C for 5 min, (3) 35 cycles of 94 °C for 30 s, 55 °C for 30 s (annealing temperature depending on the primers used), and 72 °C for 30 s, (4) finally extended at 72 °C for 5 min, and (5) stored at 4 °C. The amplified products were visualized by non-denaturing polyacrylamide gel electrophoresis at 220 V for 90 min. After rinsing, silver staining, and development, the gel was imaged using a Bio-Rad gel detection system (Bio-Rad, Hercules, CA, USA).

**Table 1:** Information on 14 pairs of microsatellite primers and genetic diversity parameters of 176 *Prunus sibirica.*

Locus	Repetition Motif	Forward and reverse primer sequence (5′–3′)	Temperature (°C)	Length (bp)	*Na*	*Ne*	*Ho*	*He*	*I*	*PIC*
L25	ATGT	F:TACCAGCTAGCTATGACCCCAAAC R:ACCGAAACAACCAGATTTGATCTC	62.7 62.7	117	13.0000	6.3171	0.3693	0.8417	2.0045	0.8220
L75	TTC	F:GCTGTTTGCATTGGTCCATACTCT R:CACTCAACTTATTCATCCAGACTCCA	63.7 62.8	151	12.0000	3.2636	0.1705	0.6936	1.5161	0.6460
P3	ATG	F:AGGGCTTTCATTCCTTTAAGTTGG R:GGGAGGAGACGAGTAGGGTAGAAA	62.9 63.2	144	12.0000	8.2790	0.2727	0.8792	2.2330	0.8670
P57H	TC	F:CGCTATGGGGTAGGTTGTACATGA R:CCCAAATATTTCAGGACCACAAGA	64.2 63.3	140	9.0000	5.1554	0.0114	0.8060	1.7930	0.7780
X32H	AGC	F:TACGCTTCAAACAAGTACAGCAGC R:TGAGGCGAGAGAATAGATAAGAAGGA	62.9 62.9	150	13.0000	3.3891	0.1023	0.7049	1.6067	0.6680
X47	CAGTC	F:ATCCGAATCCGATCGATTAAGTCT R:CAAGTCCCTTCATGTTGTTCTGTG	63.3 63.0	144	19.0000	7.8084	0.6875	0.8719	2.3287	0.8590
X87	GGA	F:GGCCAGCCTCTTACTCAATAGACA R:GTCGTCTAAACACAACACCCAACA	63.0 63.3	124	13.0000	6.3921	0.3182	0.8436	2.0681	0.8270
X8H	AAT	F:GTGTTGGTGTTTGGAGGTTTTCTC R:GGGACATCCTTTAGGGTCCACTAC	63.1 63.2	123	13.0000	4.3133	0.0852	0.7682	1.8281	0.7450
Y50	CAT	F:ATATCGCACACTGCAAACACTAGC R:CGATTGCCATGGTCACTATTCTTA	62.7 62.3	160	17.0000	7.5276	0.2273	0.8672	2.2942	0.8550
Y65	GA	F:GAGAAGGAGACGAAGCTGTGAAAG R:ACGAAATAGCGTCCAGATTCAATG	63.0 63.5	159	19.0000	8.0709	0.2386	0.8761	2.4096	0.8650
L23	ATC	F:CAAATGTTGACATCTTGACGTGGT R:TTGGTCTGTATTTGTGACGTGGTT	63.3 62.7	160	13.0000	8.3392	0.2102	0.8801	2.2468	0.8680
L62	TCCTCG	F:CTGGCAATGGCATTTATGTTGTAG R:TTACCCTACCATCACCATGTAACG	62.7 62.0	147	8.0000	5.9284	0.4091	0.8313	1.8763	0.8100
P40H	GT	F:TTTGGTAAAAGACAACGACCCACT R:TCCAACTCACACCCAAGTGATAGA	63.0 63.0	155	17.0000	9.0865	0.6477	0.8899	2.4459	0.8810
Y5	AT	F:AAGGAGTGCAAGAATGAGTGAACC R:GCAAGCCTTCTTCATATAGAGCCA	63.0 63.0	148	16.0000	6.8797	0.6989	0.8546	2.2027	0.8400
Mean					13.8571	6.4822	0.3178	0.8292	2.0610	0.8093

*Na*, Number of alleles; *Ne*, Number of effective alleles; *Ho*, Observed heterozygosity; *He*, Expected heterozygosity; *I*, Shannon’s information index; *PIC*, Polymorphism information content.

### Data analysis

We used Image Lab v 6.1 software to read the gel bands and obtained the raw genotype data after manual correction. The data were converted to different formats for further analysis using GenAlEx v 6.502^36^. POPGENE v 1.32 software^37^ was used to calculate genetic diversity parameters, including the number of alleles (*Na*), the number of effective alleles (*Ne*), observed heterozygosity (*Ho*), expected heterozygosity (*He*), and Shannon’s information index (*I*)^38–40^. Polymorphic information content (*PIC*) was determined using Cervus v 3.0 software^41^.

The results of analysis of molecular variance (AMOVA), pairwise *F*_*ST*_ analysis among populations, and principal coordinate analysis (PCoA) were computed by GenAlEx v 6.502^36,42,43^. In addition, we used GenAlEx v 6.502 to mantel test the correlations about genetic distance with geographical distance and genetic distance with elevation differences^37^.

Genetic structure was analyzed based on Bayesian clustering using STRUCTURE v 2.3.4^44,45^. This software was performed by the following parameters: (1) used the admixture model to perform a Markov chain Monte Carlo (MCMC) simulation, and set at 200,000 and 100,000 corresponded to the number of repetitions and the length of the burn-in period, and (2) set the K value from 1 to 10 with 10 repetitions. The optimal value of K was estimated using Evanno’s ∆K method and Structure Harvester^46,47^. A geographical distribution diagram with the proportion of subgroups membership was plotted using ArcMap v 10.2.

We calculated genetic similarity coefficients between 176 individuals using the Simple Matching (SM) algorithm and constructed clustering relationship tree graphs using the unweighted pair group method with arithmetic means (UPGMA), both using Ntsys-pc v 2.10s^43,48^. We used the MEGA v 7 software to calculate the Nei’s genetic distances between the 10 natural populations and constructed the UPGMA clustering relationship tree^49,50^.

### Ethical statement

The plant materials used in this article did not involve disputes. Plant materials were collected in compliance with institutional, national, and international guidelines and legislation. The plant material in our collection is preserved in the National Germplasm Repository of *Prunus sibirica* in Kazuo County, Liaoning Province, China.

## Results

### Genetic diversity

The extracted DNA of 176 *P. sibirica* samples were amplified by the 14 pairs of microsatellite primers, generating a total of 194 alleles (Table 1). The *PIC* of each locus was higher than 0.5, meaning that all 14 loci were highly polymorphic. The *Na* per locus ranged from 8 (L62) to 19 (Y65 and X47), with a mean of 13.8571 alleles. The *Ne* for each locus ranged from 3.2636 (L75) to 9.0865 (P40H), with an average of 6.4822. The *Ho* varied from 0.0114 (P57H) to 0.6989 (Y5), whereas the *He* ranged from 0.6936 (L75) to 0.8899 (P40H). And the mean value of *Ho* (0.3178) was lower than that of the *He* (0.8292), suggesting a lower level of heterozygosity in *P. sibirica*. The *He* value above 0.5 reflected that *P. sibirica* population was not affected by high-intensity selection and possessed high genetic polymorphism. The *I* for each locus ranged from 1.5161 (L75) to 2.4459 (P40H), with an average of 2.0610.

The genetic diversity parameters of the 10 *P. sibirica* populations are shown in Table 2. The *Na* per population ranged from 3.6429 (R population) to 8.2143 (NZD population), with a mean of 5.8500. The *Ne* per population ranged from 2.3912 (R population) to 5.3442 (NZW population), with a mean of 3.7703. The *I* value per population ranged from 0.8769 (R population) to 1.7759 (NZW population), with a mean value of 1.3677. The average of *He* (0.6590) was higher than that of *Ho* (0.3183). These comparisons indicated that the NZW population shows the highest genetic diversity within the assessed populations, whereas the R population shows the lowest.

**Table 2:** Genetic diversity parameters for 10 natural populations of *Prunus sibirica.*

Population	*Na*	*Ne*	*Ho*	*He*	*I*
LK	7.5000	4.7555	0.3279	0.7425	1.6411
LC	5.6429	3.9840	0.3714	0.6796	1.4206
LB	8.0000	5.0340	0.3269	0.7477	1.6899
NA	4.5000	2.9232	0.3643	0.5771	1.1279
NZW	7.8571	5.3442	0.3379	0.7937	1.7759
NZD	8.2143	4.9964	0.3171	0.7750	1.7454
R	3.6429	2.3912	0.3367	0.4650	0.8769
HL	3.8571	2.6537	0.2714	0.5939	1.0750
HW	4.7143	3.0171	0.2479	0.6189	1.2065
HZ	4.5714	2.6036	0.2812	0.5968	1.1173
Mean	5.8500	3.7703	0.3183	0.6590	1.3677

LK, Kazuo Liaoning; LC, Chaoyang Liaoning; LB, Beipiao Liaoning; NA, Aohan Inner Mongolia; NZW, Wadi Inner Mongolia; NZD, Dahewan Inner Mongolia; R, Zabaykalsky Krai; HL, Luanping Hebei; HW, Weichang Hebei; HZ, Zhuolu Hebei. *Na*, Number of alleles; *Ne*, Number of effective alleles; *Ho*, Observed heterozygosity; *He*, Expected heterozygosity; *I*, Shannon’s information index.

### Genetic differentiation

AMOVA showed that 15% of the genetic variation occurred among the 10 *P. sibirica* populations and 85% within them (Table 3). The genetic differentiation among populations was low, moderate, and high when the *F*_*ST*_ value was between 0.00 and 0.05, 0.05 and 0.15, and 0.15 and 0.25, respectively, greater than 0.25 indicating a large genetic differentiation. The genetic differentiation of *P. sibirica* detected by *F*_*ST*_ was high (*F*_*ST*_ = 0.151 > 0.15, *p* < 0.01), with the gene flow of 1.401 (*Nm* > 1), which indicated a moderate interchange of genes among these populations. Paired *F*_*ST*_ values (Table S2) indicate the degree of genetic differentiation between populations, with the highest differentiated being the R and HL populations (*F*_*ST*_ = 0.415 > 0.25) and the lowest differentiated being the LB and LC populations (*F*_*ST*_ = 0.002 < 0.05).

**Table 3:** Analysis of molecular variance (AMOVA) of *Prunus sibirica.*

Source	d.f.	Sum of square	Mean of square	Variance components	Percentage of variation	Fixation index
Among populations	9	350.805	38.978	0.898	15%	*F*_*ST*_ = 0.151***
Among individuals	166	1300.766	7.836	2.806	47%	*F*_*IS*_ = 0.558***
Within individuals	176	391.500	2.224	2.224	38%	*F*_*IT*_ = 0.625***
Total	351	2043.071		5.928	100%	*Nm* = 1.401

d.f. degrees of freedom, *** significant of data rand probability, *p* < 0.001.

A UPGMA dendrogram generated based on Nei’s genetic distances of the 10 *P. sibirica* populations (Fig. S1) revealed that at a coefficient of 0.6, the *P. sibirica* populations could be classified into subgroups A and B, with subgroup B comprising populations HZ, HL, and HW, and the remaining populations grouped in subgroup A. The pairwise *F*_*ST*_ values (Table S2) within subgroup A ranged from 0.002 (LB and LC) to 0.287 (R and LC) and from 0.002 to 0.141 (NA and NZW) without the R population. The pairwise *F*_*ST*_ values within subgroup B ranged from 0.011 (HL and HW) to 0.065 (HW and HZ). With the exception of population R, which showed substantial genetic differentiation from the others, we found a moderate and low degree of genetic differentiation within subgroups (*F*_*ST*_ < 0.15).

### Genetic structure

**Figure 1:**
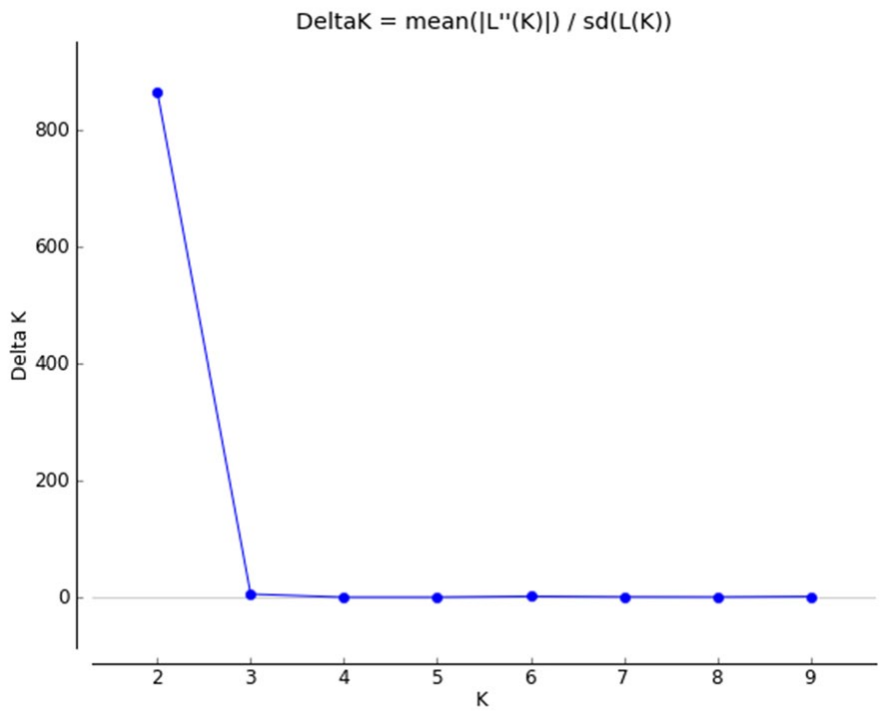
Relationship between the rational cluster K and estimated value ΔK.

**Figure 2:**
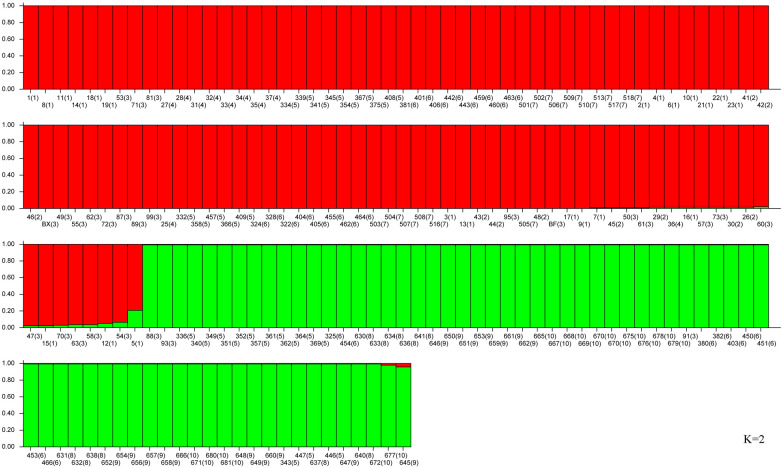
A genetic structure analysis for 176 *Prunus sibirica* individuals based on Bayesian simulation (K = 2). Red for cluster 1, and green for cluster 2. The ordinate is the Q value, the abscissa is the code of the individual and their natural population. (1), (2), (3), (4), (5), (6), (7), (8), (9), and (10) represent LK, LC, LB, NA, NZW, NZD, R, HL, HW, and HZ, respectively.

**Figure 3:**
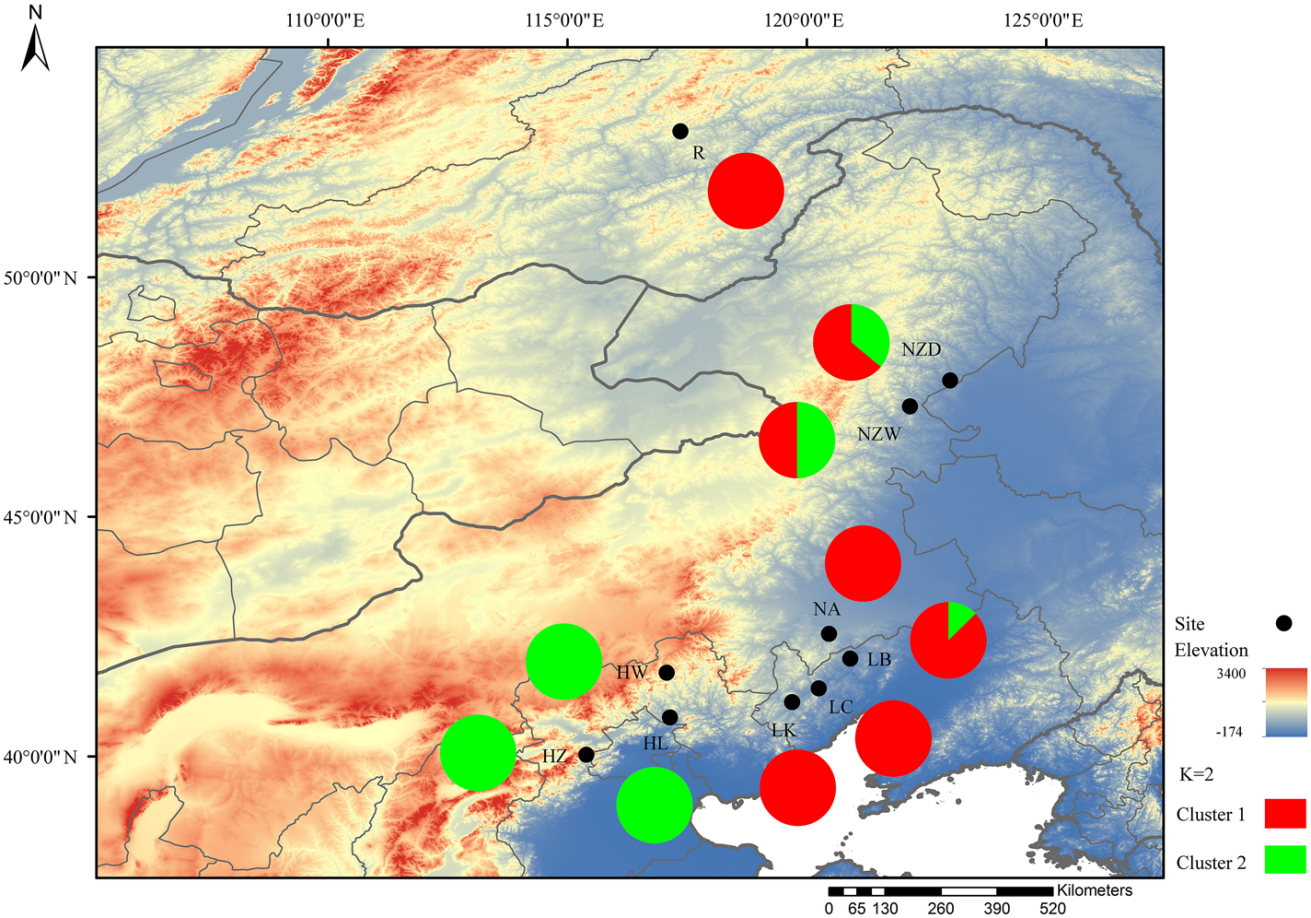
The proportions of subgroup memberships in each of the 10 *Prunus sibirica* populations.

The STRUCTURE results revealed that ∆K reached a maximum value when K = 2 (Fig. 1), thereby indicating that the 176 *P. sibirica* samples can be organized into two subgroups (clusters 1 and 2). The subgroup membership probabilities of 176 individuals from the 10 populations are shown in Fig. 2. Moreover, the proportion of subgroups membership of 10 nature populations was shown in Fig. 3. We detected significant differences in the geographical distribution of two subgroup members, dividing the natural populations into eastern and western subgroups that separately correspond to subgroups A and B. Three populations NZD, NZW, and LB showed a relatively mixed, suggesting possible gene flow between subgroups.

With the increase of K value (K = 3, 4, and 5), clusters 1 was separated into smaller subgroups based on the geographical distribution (Figs. S2–S4). According to the genetic structure analysis, the R population showed a relatively pure genetic component, and the other populations in clusters 1 contain admixed genetic components (Figs. S6–S8). When K = 6, clusters 2 was separated into two subgroups, showing a certain mixed state (Figs. S5, S9).

**Figure 4:**
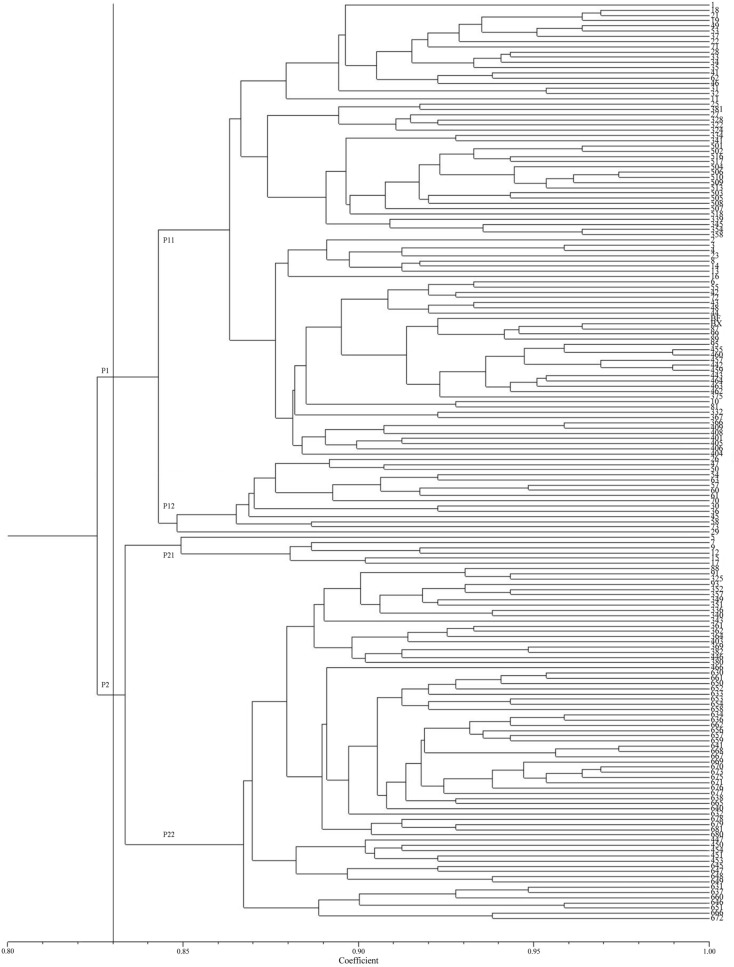
Unweighted pair group method with arithmetic means (UPGMA) cluster analysis of 176 *Prunus sibirica* individuals based on Simple Match (SM) similarity coefficient.

Similarly, the findings of PCoA (Fig. S10) showed that the 176 *P. sibirica* samples could be divided into two subgroups in complete consistency with the structure result. Furthermore, the constructed UPGMA tree clearly shows that *P. sibirica* individuals can be divided into two subgroups (P1 and P2) under a coefficient of 0.83 (Fig. 4). At a genetic similarity coefficient of 0.84, the P2 subgroup can be subdivided into P21 and P22, the latter of which was completely consistent with cluster 2 of the structure result. Mantel test revealed the genetic distance of 176 *P. sibirica* individuals correlated with geographical distance (r = 0.077, *p* < 0.01) (Fig. S11) and elevational distribution (r = 0.112, *p* < 0.01) (Fig. S12), respectively.

## Discussion

### Genetic diversity

Genetic diversity reflects the evolutionary characteristics of species and their adaptation to the environment, which is typically determined based on the data of continuous traits or discrete allelic states^51^. In this study, we obtained discrete allelic data for 176 *P. sibirica* accessions from 10 natural populations based on analysis using 14 microsatellite markers. We evaluated the genetic diversity of *P. sibirica* in terms of allelic richness, heterozygosity, and allelic diversity. Compared with previous studies^11–15,17^, the present study significantly extends the sampling area of *P. sibirica* to the north (Zabaykalsky Krai), with a corresponding expansion of climate type from intermediary and warm to cold temperate zones. The mean genetic parameters of each locus detected in our research (*Na* = 13.857, *He* = 0.829, and *I* = 2.061) is comparable to that reported by Wang et al.^15^ (*Na* = 19.323, *He* = 0.774, and *I* = 2.062). These findings indicate that within the genus *Prunus*, *P. sibirica* has a slightly higher level of genetic diversity than apricot (*Prunus armeniaca* L.) (*Na* = 24.360, *He* = 0.732, and *I* = 1.837)^21^, and a significantly higher diversity compared with sweet cherry (*Prunus avium* L.) (*Na* = 9.800 and *He* = 0.700)^52^, peach (*Prunus persica* L.) (*Na* = 6.410 and *He* = 0.490)^53^ and alpine plum (*Prunus brigantina* Vill.) (*Na* = 5.040 and *He* = 0.430)^54^. Moreover, compared with xylophyta in the Rosaceae family, the diversity is lower than that of apple (*Malus* × *domestica* Borkh.) (*Na* = 23.060 and *He* = 0.830)^55^ and higher than that of pear (*Pyrus pyrifolia*) (*Na* = 7.120 and *He* = 0.780)^56^. The average genetic parameters of the populations detected in our research (*Na* = 5.850, *He* = 0.659, and *I* = 1.368) is higher than that of *Prunus mira* Koehne (*Na* = 3.800, *He* = 0.520, and *I* = 0.950)^57^. We speculate that this relatively high level of genetic diversity in *P. sibirica* is related to their area of distribution. Li et al.^13^ considered that *P. sibirica* might accumulate considerable intraspecific genetic variation to adapt to the diverse ecological conditions in its wide range of distribution. Climatic fluctuations over recent millions of years have tended to influence the genetic diversity of species. However, the center of *P. sibirica* distribution is East Asia, which is generally considered to have been a large-scale refuge during the Pleistocene, characterized by a relatively small range of environmental changes^58^. We thus speculate that this species may have retained a large proportion of its intraspecific genetic variation. Furthermore, Mehlenbacher et al.^1^ found that the hybridizations between any two true apricot species were successful, including those among *P. sibirica*, *P. armeniaca*, *P. mandshurica*, and *P. mume*. Thus, interspecific hybridization may also influence the accumulation of genetic variation in *P. sibirica*.

In the present study, we found that the natural populations of *P. sibirica* distributed in different geographical regions differ significantly in terms of genetic diversity, with the highest levels of genetic diversity being detected in the NZW and NZD populations distributed in the northeastern region of Inner Mongolia, followed by the LB, LK, and LC populations in western Liaoning, and then the HW, HZ, and HL populations in northern Hebei and the NA population in central Inner Mongolia, with the lowest diversity detected in R populations in southern Zabaykalsky Krai. The natural habitat of the NZW and NZD populations is located near the Changbai Mountains overlaps with the natural distribution range of the Manchurian apricot (*Prunus mandshuria* Skv.), and most germplasms are older and less disturbed by human activities, which may account for higher genetic diversity of these populations. Population R is the only population distributed within the cold temperate zone, and although has lower genetic diversity than other assessed populations, it has accumulated unique genotypes during the process of adapting to its marginal habitats. In addition, germplasm resources in western Liaoning have been widely reported^12–15^. In the present study, the ranking of the genetic diversity levels in the Liaoning populations (LC < LK < LB) is consistent with the previous findings^12,15^. Contrastingly, Li et al.^13^ were of opinion that the genetic diversity was higher in the LC population than in the LK population. However, we found the genetic diversity of the NA population with similar geographical distance was relatively low, which is also consistent with the results presented by Chen et al.^12^ and Wang et al.^15^.

### Genetic differentiation and genetic structure

Woody plants tend to maintain a larger extent of variation within populations than between populations, particularly in the case of those species with a large geographical range, outcrossing, and seed dispersal mediated mainly by wind or animals^59^. This is consistent with our present findings for *P. sibirica*, the genetic variation of which mainly exists within species and populations. We detected a high degree of genetic differentiation at the species level (0.15 < *F*_*ST*_ < 0.25), and given because the existing gene flow (*Nm* = 1.401 > 1), there is a resistance to genetic drift, there was no further differentiation^43,60^. Consequently, the genetic structure was relatively stable. Given the extensive and discontinuous distribution of *P. sibirica*, we speculate that gene flow among populations is maintained by animal-mediated seed dispersal rather than a combination of pollen transmission and insect- and wind-mediated dispersal, which is also consistent with the views of Li et al.^13^. In the distribution area surveyed in the present study, rodents hoarded *P. sibirica* seeds at dispersed locations, and exploited these less often or consumed them only in the absence of alternatives, owing to the amygdalin content^61^. We speculate that the primary mode of gene flow is the moderate exchange of genes between the buried *P. sibirica* seeds and local populations through pollination after they have developed to reproductive maturity.

We established that the genetic structure of *P. sibirica* consists of two subgroups: the eastern and western subgroups, based on the STRUCTURE, clustering, and PCoA results. We found that whereas there was a moderate and low genetic differentiation among populations within the same subgroup (excepting the R population), there was a high differentiation between the populations belonging to different subgroups, among which the R population of the eastern subgroup and the HL population of the western subgroup were characterized by the highest genetic differentiation (*F*_*ST*_ = 0.415 > 0.25). Based on the common natural hybridization phenomena among plants of the Li subgenus apricot group^1^ and the distribution of plants of subgenus apricot in each region, we speculate that western subgroup may have undergone an introgression with *P. armeniaca*, which has thus influenced the genetic structure of the *P. sibirica*. Liu et al.^14^ divided *P. sibirica* germplasm into two subgroups and similarly believed that the northwestern subgroup was largely derived as a consequence of introgression between the northeastern subgroup and *P. armeniaca*. Mantel test results revealed the significant influence of geographical distance and elevation distribution in contributing to the genetic structure among *P. sibirica* in the study area (*p* < 0.001). Furthermore, the area surveyed in the present study falls within a semi-humid region, and given the high degree of genetic differentiation between populations in the western subgroups region lying in the warm temperate zone and those in the eastern subgroup region lying in the middle and cold temperate zone, we speculate that temperature also influences the genetic structure of *P. sibirica*.

### Conservation remarks

As a consequence of frequent human activities, such as fruit and seed picking, grazing, and deforestation, large areas of *P. sibirica* habitat have been destroyed, and its natural distribution range continues to decline^13^. To maximize the conservation of *P. sibirica* genetic resources, we have developed conservation strategies for *P. sibirica* in the study area depending on our findings. The genetic variation of *P. sibirica* exists occurs mainly within populations, and thus conservation efforts should focus on maintaining and increasing the genetic diversity level within populations. In this regard, we established that the genetic diversity of the NZW and NZD populations distributed in northern Inner Mongolia was significantly higher than that of other assessed populations, and thus the in situ conservation of these two populations should be given priority^22^. To protect the existing populations, we recommend the establishment of protected areas or sites to enhance management, which would require cooperation with the local government in charge of forest management. Moreover, to protect the genetic resources of populations with relatively low levels of genetic diversity, we propose to improve the habitats of *P. sibirica* via complementary silvicultural actions using forest plants from the same or genetically related populations. Furthermore, we have collected genetic materials from the natural population and ex situ preserved in a germplasm conservation base. Lay a solid foundation basis for long-term breeding and genetic resource conservation of *P. sibirica*.

## Conclusions

In this research, we used 14 microsatellite markers to analyze samples collected from 176 individuals in 10 natural populations of *Prunus sibirica* to assess their genetic diversity, differentiation, and structure. Our findings indicate that *P. sibirica* has a high level of genetic diversity, particularly within the NZW and NZD populations distributed in northeast Inner Mongolia. At the species level, there is a high degree of genetic differentiation and a relatively stable genetic structure. The genetic structure of *P. sibirica* can be affected by geographical distance and elevation distribution, with 10 natural populations dividing into two subgroups. This finding will contribute to the conservation research and the rational utilization of *P. sibirica*.

## Supplementary Information

Supplementary Information.

## Data Availability

The datasets generated during and analyzed during the current study are available from the corresponding author on reasonable request.
