# Supplementary material for: Genetic diversity and conservation of Siberian apricot (Prunus sibirica L.) based on microsatellite markers
Source: Sci Rep. 2023 Jul 11;13:11245. doi: 10.1038/s41598-023-37993-2 (PMC10336122; doi:10.1038/s41598-023-37993-2)
Supplement: Supplementary file 1 — Supplementary Information. [file 41598_2023_37993_MOESM1_ESM.pdf]

Online resources: *Scientific Reports*

Genetic diversity and conservation of Siberian apricot (*Prunus sibirica* L.) base on microsatellite markers

Xinxin Wang [1, 2], Li Wang [1, 2], Yongqiang Sun [1, 2], Jianhua Chen [1, 2], Quangang Liu [1, 2], Shengjun Dong [1,2] *

[1] College of Forestry, Shenyang Agricultural University, Shenyang, 110866, Liaoning, China
[2] Key Laboratory for Silviculture of Liaoning Province, Shenyang, 110866, Liaoning, China
* Correspondence authors. E-mail addresses: dsj928@163.com (S. Dong).

Table S1 Geographical information of 176 *P. sibirica* individuals from 10 populations

Population	ID	Sample size	Code	Latitude (◦N)	Longitude (◦E)	Elevation (m)
Kazuo, Liaoning	LK	22	1	41.060170	119.852926	343
			2	41.060177	119.852702	343
			3	41.057379	119.854049	346
			4	41.054727	119.854157	346
			5	41.012958	119.781834	361
			6	41.013952	119.781727	361
			7	41.013217	119.781601	361
			8	41.014809	119.792973	372
			9	41.018184	119.793405	393
			10	41.060682	119.785585	408
			11	41.061443	119.787027	408
			12	41.179814	119.615164	358
			13	41.179651	119.618039	359
			14	41.178538	119.616817	359
			15	41.177832	119.615452	358
			16	41.177995	119.617644	359
			17	41.223412	119.515809	536
			18	41.221133	119.516384	536
			19	41.221547	119.513716	536

Population	ID	Sample size	Code	Latitude (◦N)	Longitude (◦E)	Elevation (m)
			21	41.286382	119.563643	651
			22	41.283942	119.568387	699
			23	41.282980	119.602055	691
Chaoyang, Liaoning	LC	10	26	41.446044	120.239149	248
			29	41.445865	120.239356	248
			30	41.445767	120.239432	248
			41	41.379036	120.278567	365
			42	41.445894	120.239525	248
			43	41.367424	120.260349	415
			44	41.371511	120.297647	556
			45	41.445848	120.239607	248
			46	41.375653	120.294988	509
			48	41.441108	120.184584	222
Beipiao, Liaoning	LB	26	47	42.035995	120.910047	276
			49	42.035925	120.909378	276
			50	42.036009	120.909293	276
			53	42.036467	120.909769	276
			54	42.036457	120.909329	276
			55	42.036494	120.909050	276
			57	42.036451	120.908498	276
			58	42.036444	120.908947	276
			60	42.036417	120.909526	276
			61	42.036434	120.909957	276
			62	42.036434	120.910546	276
			63	42.036417	120.910779	276
			70	42.036357	120.908358	276

Population	ID	Sample size	Code	Latitude (◦N)	Longitude (◦E)	Elevation (m)
			71	42.042072	120.908875	283
			72	42.036317	120.908857	276
			73	42.036110	120.908800	276
			81	42.033159	120.895535	279
			87	42.040090	120.906647	288
			88	42.045194	120.910567	283
			89	42.034974	120.907761	288
			91	42.045269	120.908497	283
			93	42.045289	120.908828	283
			95	42.040385	120.910636	276
			99	42.039769	120.912971	276
			BF	42.033849	120.901796	288
			BX	42.033367	120.906467	288
Aohan, Inner Mongolia	NA	10	25	42.553323	120.466004	423
			27	42.553528	120.465025	423
			28	42.553163	120.463776	423
			31	42.553356	120.464755	423
			32	42.553376	120.464387	423
			33	42.553402	120.464432	423
			34	42.553874	120.463857	423
			35	42.554790	120.464526	423
			36	42.554594	120.463947	423
			37	42.554440	120.463900	423
Wadi, Inner Mongolia	NZW	26	332	47.311528	122.144083	470
			334	47.310194	122.144528	470

Population	ID	Sample size	Code	Latitude (◦N)	Longitude (◦E)	Elevation (m)
			336	47.312250	122.141444	476
			339	47.310556	122.145056	470
			340	47.310333	122.145167	470
			341	47.309694	122.145083	470
			343	47.310000	122.145583	470
			345	47.309778	122.144667	470
			349	47.312111	122.140167	476
			351	47.312028	122.140028	476
			352	47.311028	122.143528	470
			354	47.310389	122.143028	470
			357	47.312028	122.140417	476
			358	47.312861	122.141389	476
			361	47.311667	122.143583	470
			362	47.311444	122.143444	470
			364	47.317972	122.247194	608
			366	47.317500	122.246861	608
			367	47.317694	122.247056	608
			369	47.249500	122.128889	415
			375	47.248250	122.152861	398
			408	47.273806	122.190694	364
			409	47.273833	122.190778	364
			446	47.876583	122.941889	415
			447	47.310000	122.145194	470
			457	47.309944	122.145361	470
Dahewan, Inner Mongolia	NZD	25	322	47.821972	123.019833	319
			324	47.822639	123.018139	319

Population	ID	Sample size	Code	Latitude (◦N)	Longitude (◦E)	Elevation (m)
			325	47.823528	123.016861	319
			328	47.823444	123.016528	318
			380	47.822194	123.019083	319
			381	47.821500	123.020556	319
			382	47.822806	123.017306	319
			401	47.822000	123.019278	319
			403	47.822306	123.019722	319
			404	47.822167	123.019639	319
			405	47.823306	123.017889	319
			406	47.967528	122.903083	375
			442	47.822056	123.019056	319
			443	47.875333	122.945139	364
			450	47.876333	122.946250	364
			451	47.967222	122.903056	375
			453	47.823389	123.016583	318
			454	47.822194	123.019500	319
			455	47.823194	123.017500	319
			459	47.875417	122.945083	364
			460	47.821861	123.019361	319
			462	47.822306	123.019667	319
			463	47.823056	123.018361	319
			464	47.822167	123.018917	319
			466	47.876583	122.941889	364
Zabaykalsky Krai	R	14	501	53.097896	117.520379	714
			502	53.091380	117.548996	672
			503	53.087821	117.576059	654

Population	ID	Sample size	Code	Latitude (◦N)	Longitude (◦E)	Elevation (m)
			504	53.079790	117.570590	676
			505	53.063307	117.589201	822
			506	53.055877	117.564948	938
			507	53.050105	117.546283	939
			508	53.046842	117.517194	932
			509	53.053131	117.493882	814
			510	53.076792	117.474288	683
			513	52.991522	116.936739	835
			516	52.964459	116.970742	731
			517	52.942795	116.921258	762
			518	52.959207	116.841971	683
Luanping, Hebei	HL	10	630	40.816672	117.179761	463
			631	40.816739	117.179792	463
			632	40.816886	117.179892	463
			633	40.817042	117.180139	463
			634	40.817022	117.180153	463
			636	40.816858	117.179464	463
			637	40.816961	117.179417	463
			638	40.816772	117.179556	463
			640	40.788853	116.991017	479
			641	40.788839	116.990131	479
Weichang, Hebei	HW	17	645	41.745672	117.069278	1092
			646	41.745917	117.069239	1092
			647	41.745964	117.066889	1092
			648	41.745914	117.068661	1092
			649	41.745878	117.068456	1092

Population	ID	Sample size	Code	Latitude (◦N)	Longitude (◦E)	Elevation (m)
			650	41.745611	117.068047	1092
			651	41.746133	117.068628	1092
			652	41.746197	117.068833	1092
			653	41.746172	117.068861	1092
			654	41.746575	117.069044	1092
			656	41.737475	117.083367	1210
			657	41.737425	117.083178	1160
			658	41.737381	117.083089	1160
			659	41.736175	117.078753	1160
			660	41.736386	117.078492	1160
			661	41.736439	117.076869	1160
			662	41.736167	117.075892	1160
Zhuolu, Hebei	HZ	16	665	40.033050	115.413072	1224
			666	40.035103	115.412764	1292
			667	40.035108	115.412717	1292
			668	40.035222	115.412836	1292
			669	40.030833	115.394050	1229
			670	40.030950	115.394089	1229
			671	40.030969	115.394094	1229
			672	40.031000	115.394131	1229
			673	40.030992	115.393997	1229
			675	40.031136	115.394528	1229
			676	40.030783	115.393956	1229
			677	40.029639	115.389697	1249
			678	40.029600	115.389778	1249
			679	40.029606	115.389492	1249

Population	ID	Sample size	Code	Latitude (◦N)	Longitude (◦E)	Elevation (m)
			680	40.029478	115.386239	1249
			681	40.029317	115.386078	1249

Table S2 The matrix of pairwise F_{ST} among populations

Population	LK	LC	LB	NA	NZW	NZD	R	HL	HW	HZ
LK										
LC	0.068									
LB	0.058	0.002								
NA	0.106	0.076	0.077							
NZW	0.077	0.090	0.056	0.141						
NZD	0.077	0.057	0.039	0.103	0.029					
R	0.210	0.281	0.214	0.287	0.190	0.219				
HL	0.226	0.235	0.182	0.320	0.112	0.141	0.415			
HW	0.237	0.253	0.197	0.319	0.122	0.139	0.401	0.011		
HZ	0.248	0.257	0.204	0.335	0.123	0.159	0.410	0.023	0.065	

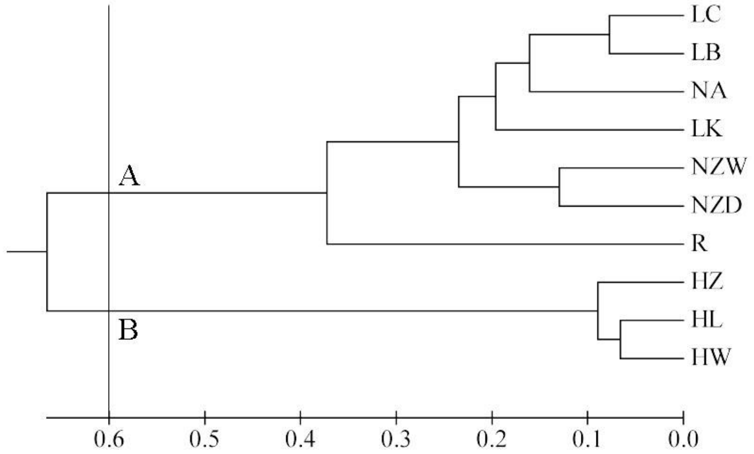

Fig.S1 A dendrogram of *Prunus sibirica* populations using unweighted pair group method with arithmetic means (UPGMA)

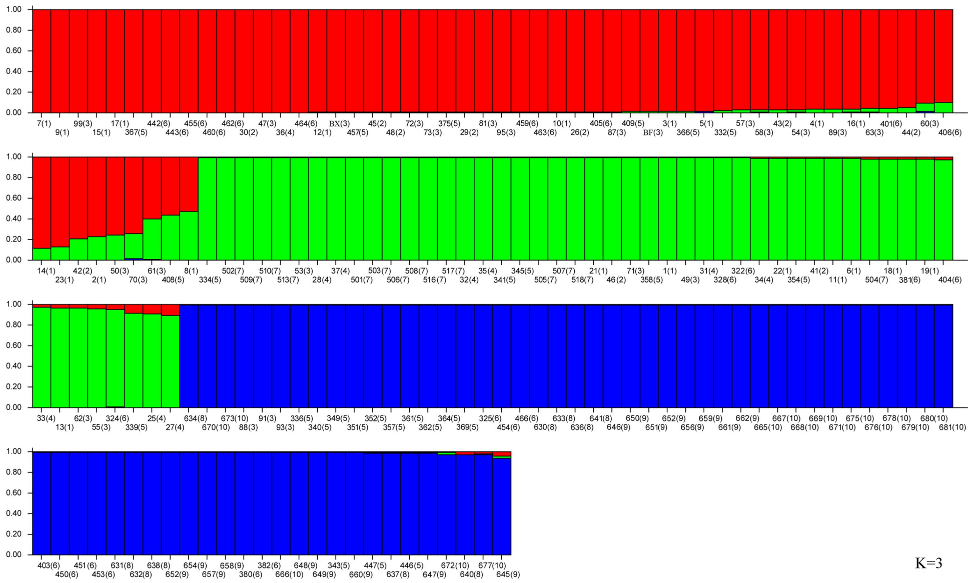

Fig.S2 A genetic structure analysis for 176 *Prunus sibirica* individuals based on Bayesian simulation (K = 3). Different colors represent different components. The ordinate is the Q value, the abscissa is the code of the individual and their natural population. (1), (2), (3), (4), (5), (6), (7), (8), (9), and (10) represent LK, LC, LB, NA, NZW, NZD, R, HL, HW, and HZ, respectively

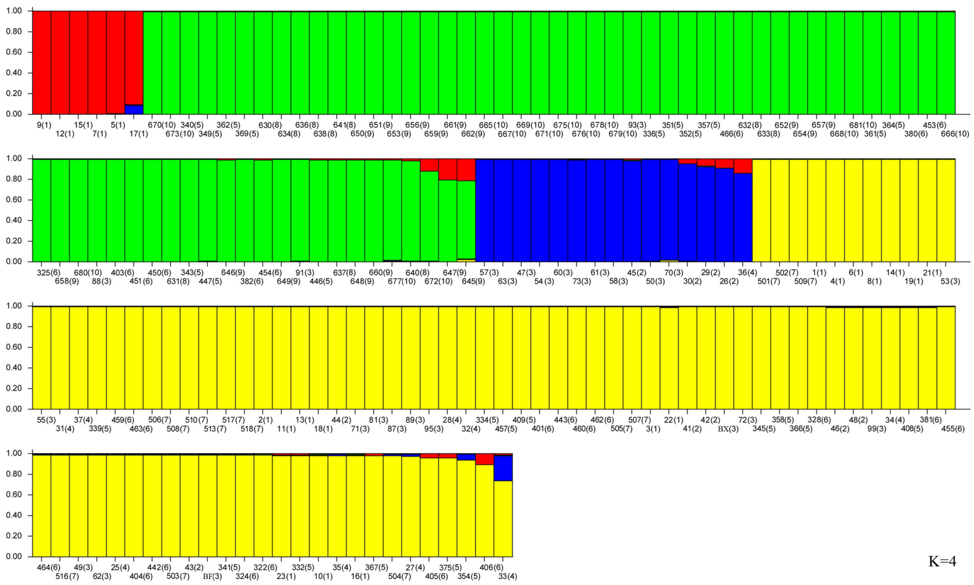

Fig.S3 A genetic structure analysis for 176 *Prunus sibirica* individuals based on Bayesian simulation (K = 4). Different colors represent different components. The ordinate is the Q value, the abscissa is the code of the individual and their natural population. (1), (2), (3), (4), (5), (6), (7), (8), (9), and (10) represent LK, LC, LB, NA, NZW, NZD, R, HL, HW, and HZ, respectively

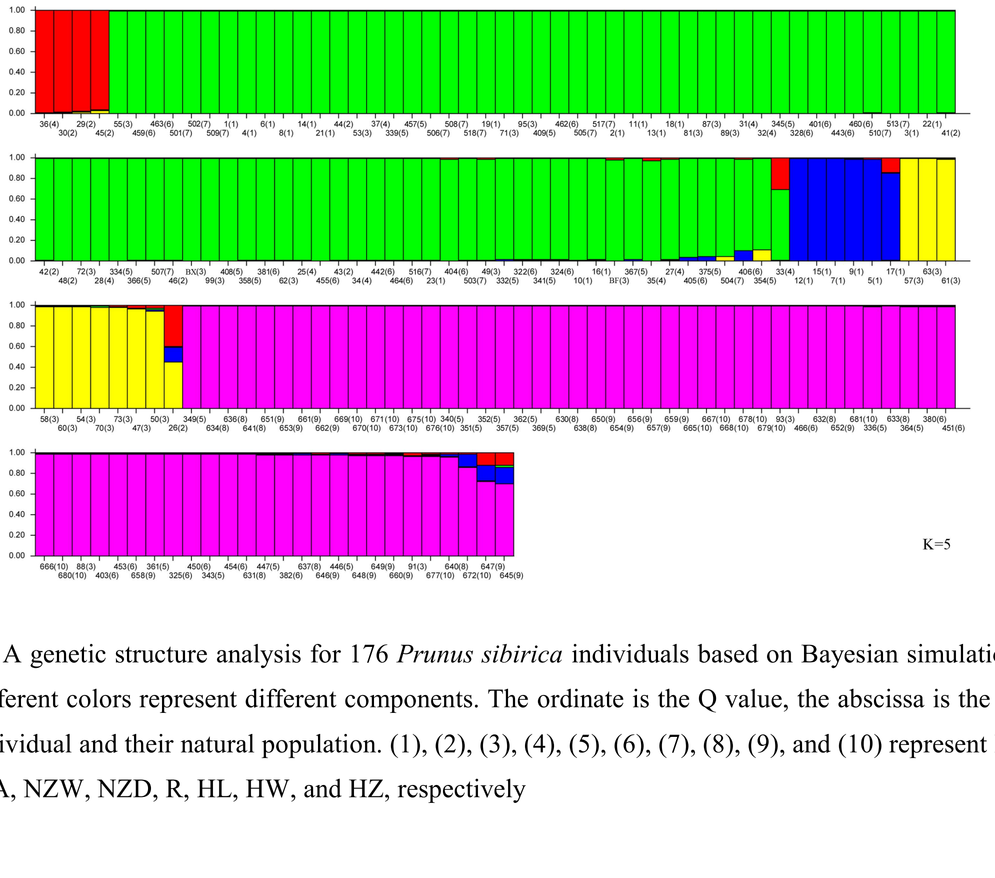

Fig.S4 A genetic structure analysis for 176 *Prunus sibirica* individuals based on Bayesian simulation (K = 5). Different colors represent different components. The ordinate is the Q value, the abscissa is the code of the individual and their natural population. (1), (2), (3), (4), (5), (6), (7), (8), (9), and (10) represent LK, LC, LB, NA, NZW, NZD, R, HL, HW, and HZ, respectively

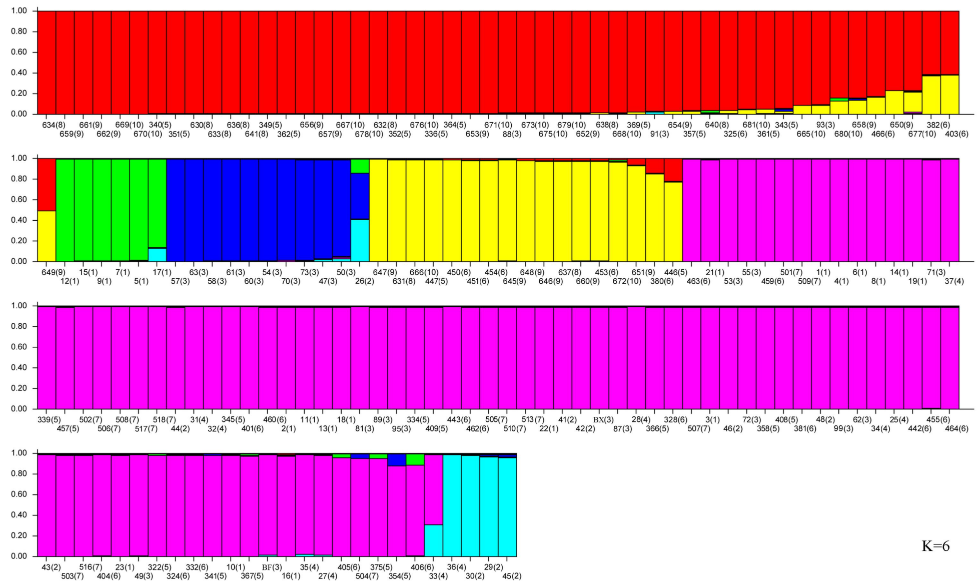

Fig.S5 A genetic structure analysis for 176 *Prunus sibirica* individuals based on Bayesian simulation (K = 6). Different colors represent different components. The ordinate is the Q value, the abscissa is the code of the individual and their natural population. (1), (2), (3), (4), (5), (6), (7), (8), (9), and (10) represent LK, LC, LB, NA, NZW, NZD, R, HL, HW, and HZ, respectively

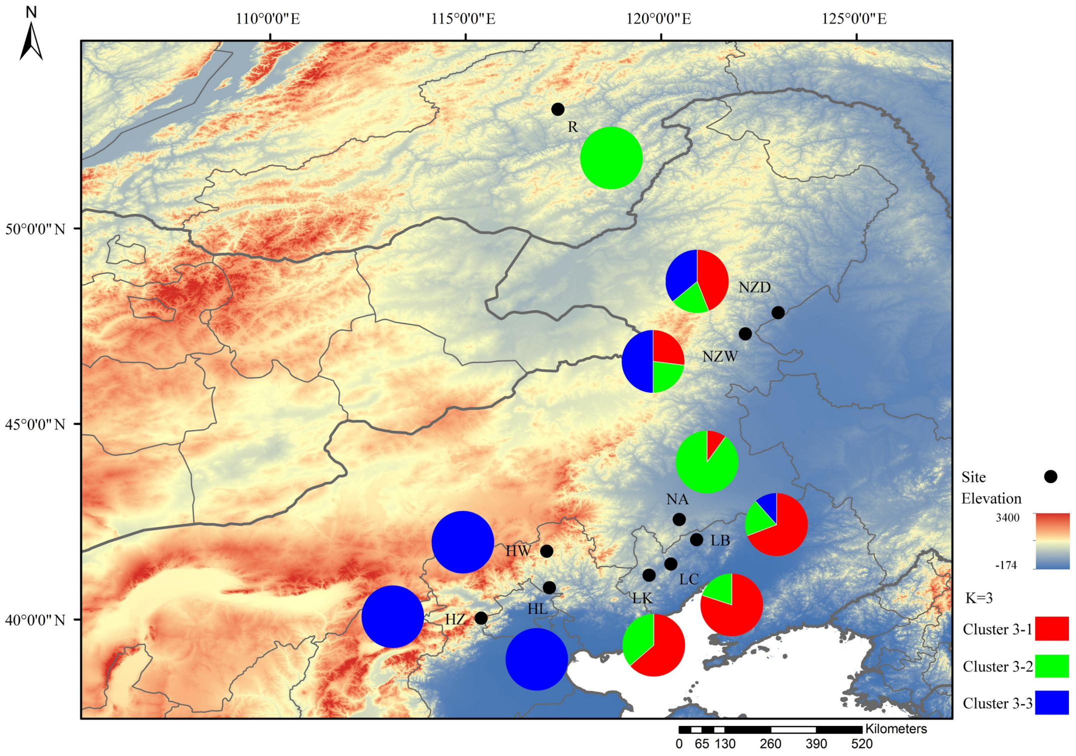

Fig.S6 The proportions of subgroup memberships in each of the 10 *Prunus sibirica* populations (K = 3).

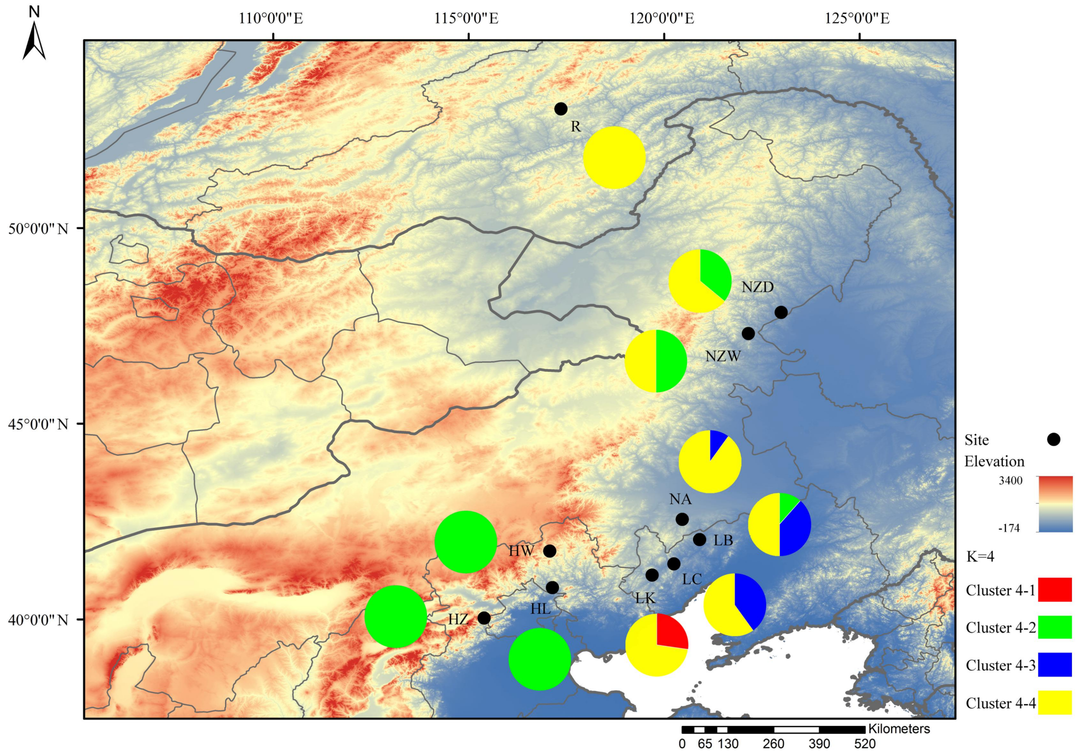

Fig.S7 The proportions of subgroup memberships in each of the 10 *Prunus sibirica* populations (K = 4).

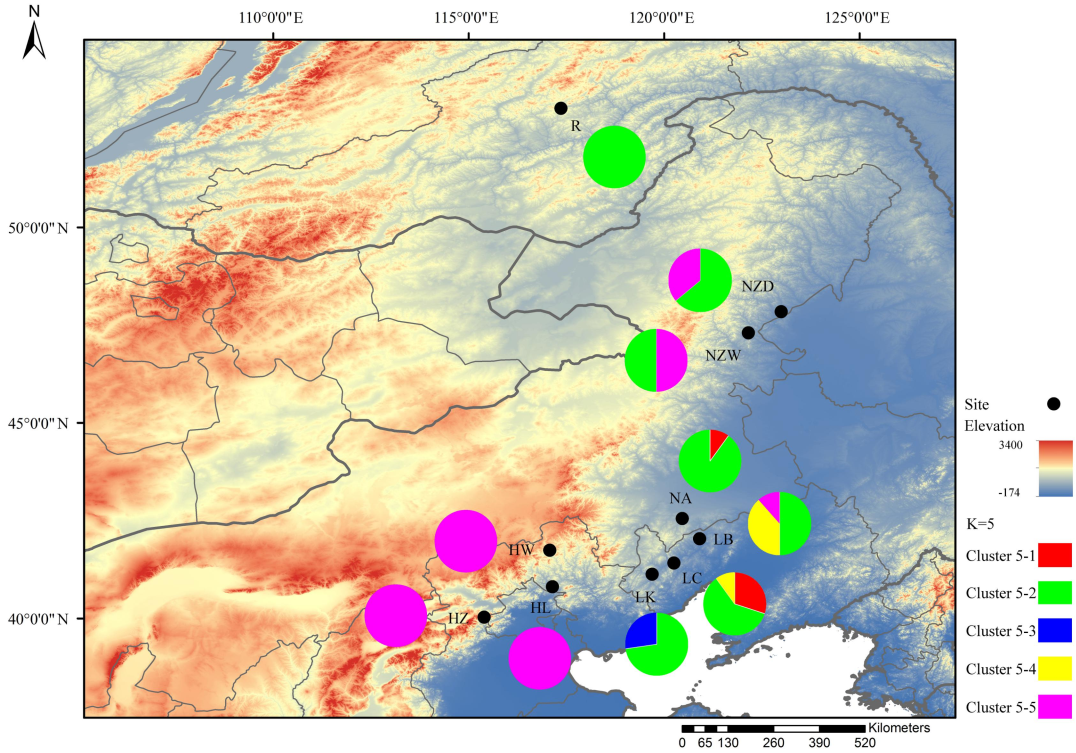

Fig.S8 The proportions of subgroup memberships in each of the 10 *Prunus sibirica* populations (K = 5).

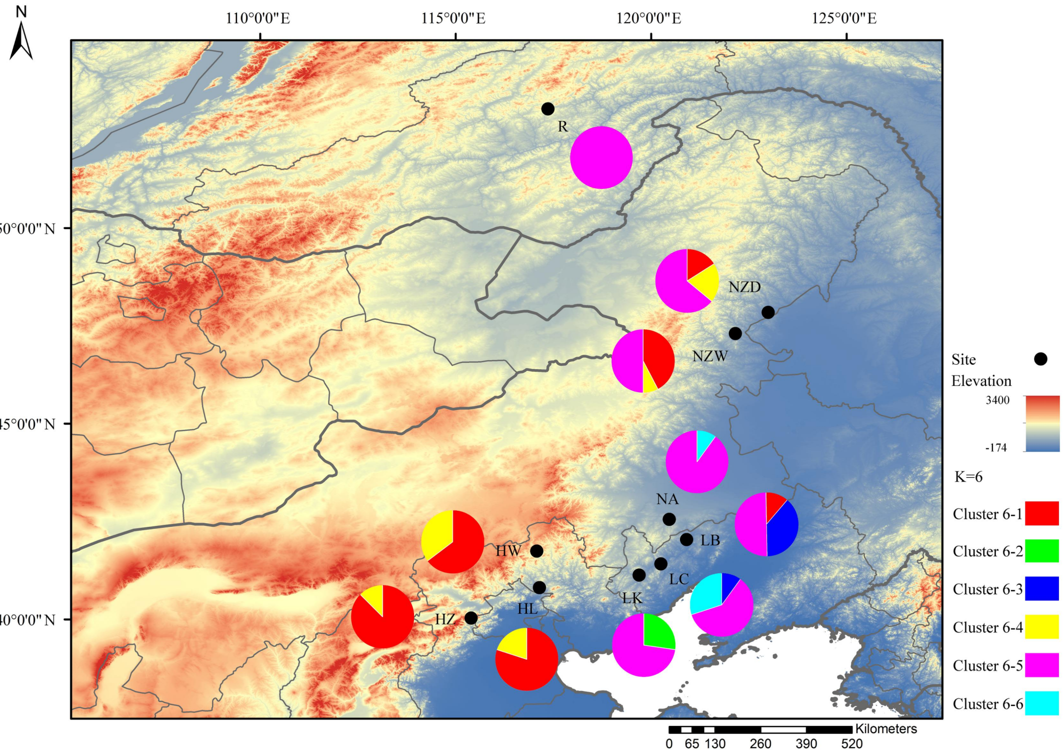

Fig.S9 The proportions of subgroup memberships in each of the 10 *Prunus sibirica* populations (K = 6).

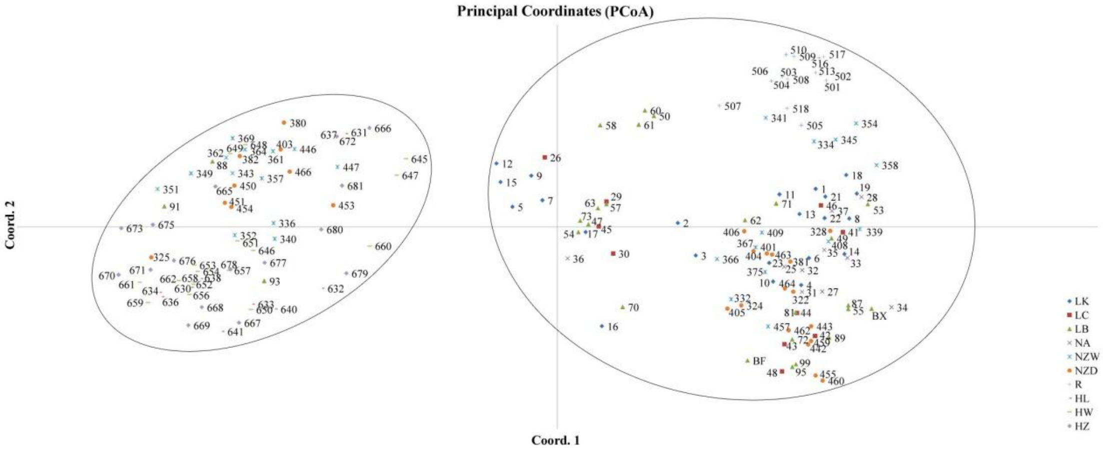

Fig.S10 Principal coordinate analysis (PCoA) of 176 *Prunus sibirica* individuals

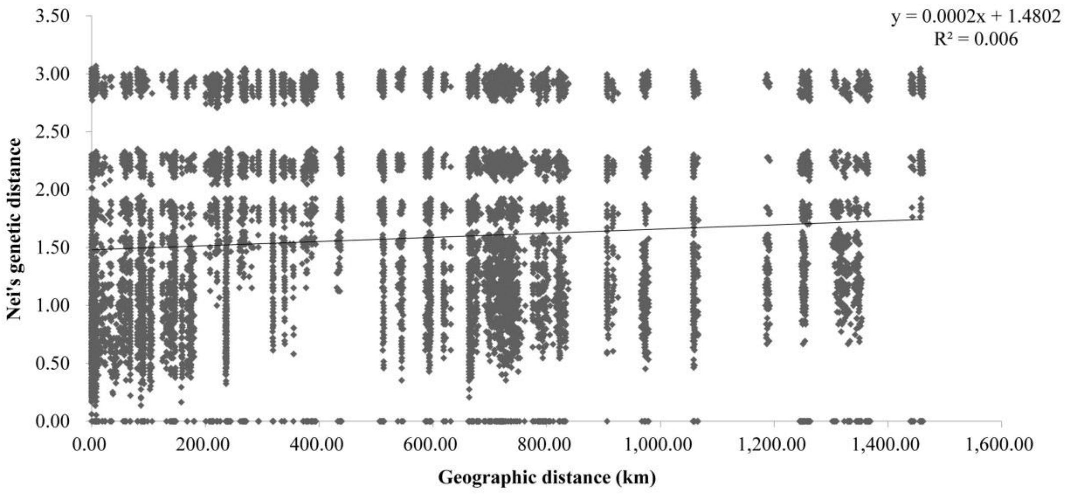

Fig.S11 Mantel test between genetic distance and geographic distance in 176 *Prunus sibirica* individuals

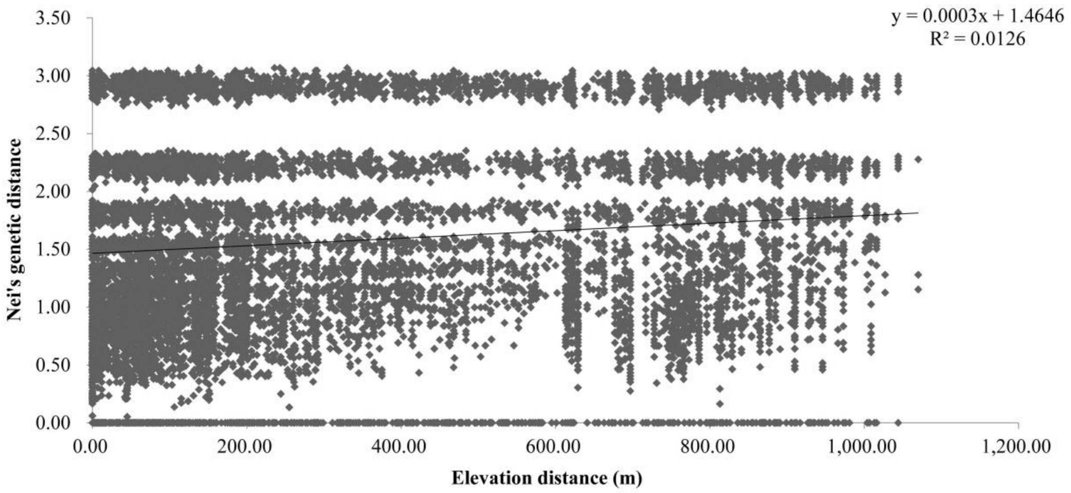

Fig.S12 Mantel test between genetic distance and elevation distribution in 176 *Prunus sibirica* individuals
